# A mindfulness-based intervention adapted to dementia caregivers: A study protocol for a randomized clinical control trial

**DOI:** 10.3389/fpsyg.2022.1062452

**Published:** 2022-12-20

**Authors:** Rea Antoniou, Despoina Georgakopoulou Toli, Hannah Lerner, Patrick Callahan, Roger Coble, Bailey Ortiz, Alissa Bernstein Sideman, Suzanne M. Shdo, Robert W. Levenson, Nuno Ferreira, Judith T Moskowitz, Katherine P. Rankin

**Affiliations:** ^1^Department of Neurology, Memory and Aging Center, University of California San Francisco, San Francisco, CA, United States; ^2^Philip R. Lee Institute for Health Policy Studies, University of California San Francisco, San Francisco, CA, United States; ^3^Department of Humanities & Social Sciences, University of California San Francisco, San Francisco, CA, United States; ^4^Global Brain Health Institute, University of California San Francisco, San Francisco, CA, United States; ^5^Department of Psychology, University of California Berkeley, Berkeley, CA, United States; ^6^Department of Social Sciences, School of Humanities and Social Sciences, University of Nicosia, Nicosia, Cyprus; ^7^Department of Medical Social Sciences, Northwestern University Feinberg School of Medicine, Chicago, IL, United States

**Keywords:** mindfulness, dementia caregiving, clinical trial, mindfulness based intervention, relational wellbeing, psychological wellbeing, mixed method design

## Abstract

Dementia caregiving, besides encompassing various challenges in tandem to the diagnosis of the care recipient, is associated with decreased psychological well-being and mental health. Accordingly, caregivers’ wellbeing has an impact on the quality of care they provide and on the relationship quality with the person in their care. The aim of the present study is to examine the effectiveness of a mindfulness-based intervention on relational and psychological wellbeing, tailored to the needs of dementia caregivers. This clinical trial (NCT04977245) will apply a randomized controlled mixed method design. Caregivers will be randomly allocated to either the mindfulness intervention or the active control group. The intervention arm is based on experiential learning and is targeted to promote caregivers’ well-being and empowerment. Assessments will include, standardized self-report questionnaires, task performance measures, and qualitative measures. All assessments will be held at three time points (baseline; t0, 0 months, post-intervention; t1, 2 months, and after maintenance; t2, 3 months) focused on three core domains (1. relational well-being, 2. psychological well-being, and 3. dementia patient’s lifestyle/activities). The primary outcome will be relational well-being, and data will be analyzed using linear mixed modelling.

## Introduction

While caring for someone with dementia can have many positive personal and relational outcomes, it can also be a very challenging task encompassing various practical and psychological demands (Quinn et al., 2009; Sörensen and Conwell, 2011; Fontaine et al., 2016; Li et al., 2016). Common adverse experiences for caregivers include over-engagement in caregiving tasks, significantly limited discretionary time, reduced access to socioemotional support, and complications related to finances and employment (Connell et al., 2001; Bertrand et al., 2006). In parallel with those challenges, caregivers often experience difficulties with mental health and psychological and relational well-being (Rabins et al., 1982; Bledin et al., 1990; Pinquart and Sörensen, 2003, 2006), which can manifest as burden, depressive symptoms, negative affect, social isolation, as well as decreased relationship quality between themselves and their care recipient (Wagner et al., 1997; Quinn et al., 2009). Caregiving demands often sustain over time and can engender significantly maladaptive psychological processes. For instance, rumination can prolong intensified feelings of negativity toward one’s role as a caregiver (Aldao et al., 2010; Guendelman et al., 2017), while criticism and emotional over-involvement with the care recipient reflect poor relationship quality and predict caregivers’ strain levels (Morris et al., 1988; Fearon et al., 1998). Caregivers who associate the quality of the relationship with their care recipient with intensified negative emotions (e.g., guilt, anger, grief) are more likely to exhibit poor coping skills (Lea Steadman et al., 2007; Shaffer et al., 2007; Cooper et al., 2010a,b; Manteau-Rao and Barrows, 2016).

Caregivers of dementia patients face additional specialized challenges that differ in tandem with the particular diagnosis and behaviors exhibited by the care recipient (Yeager et al., 2010; Leggett et al., 2011; Wong et al., 2012; George et al., 2020). Hence, a need for effective interventions to assist dementia caregiving challenges naturally emerges. While Alzheimer’s disease (AD) patients exhibit memory and cognitive dysfunction, patients with frontotemporal dementia (FTD) primarily exhibit emotional bluntness, apathy, and behavioral disinhibition (e.g., outbursts, inappropriate, or risky behaviors; Pinquart and Sörensen, 2004, 2007). Accordingly, compared to AD caregivers, FTD caregivers, and particularly those whose patient is diagnosed with the behavioral variant of FTD (bvFTD), experience significantly higher distress and strain levels, more depressive symptoms, lower perceived control, greater burden, and poorer satisfaction with the care recipient and themselves (de Vugt et al., 2006; Riedijk et al., 2006; Boutoleau-Bretonnière et al., 2008; Ascher et al., 2010). Other neurodegenerative conditions in which early behavioral changes occur, like Huntington’s disease (HD), create important challenges for family caregivers, including compromised quality of life and difficulties in communication (Hartelius, 2010; Aubeeluck et al., 2011).

Past research examining psychosocial interventions for dementia caregivers (e.g., support groups, psychoeducation, counseling) reveals inconsistent evidence for their effectiveness in decreasing burden and depression (Cooke et al., 2001; Dam et al., 2016; Collins and Kishita, 2018). However, early evidence suggests that mindfulness-based stress reduction (MBSR) programs may be effective for dementia caregivers. Mindfulness training *via* the MBSR program cultivates skills of non-reactivity, acceptance, and awareness, which may work against reactive coping mechanisms in caregivers, ultimately enhancing attributes connected to psychological well-being (Kabat-Zinn, 1992; Killingsworth and Gilbert, 2010; Lindsay and Creswell, 2017). Mindfulness may additionally impact downstream emotion regulation processes that introduce flexibility in generating cognitive appraisals, thereby facilitating savoring of positive experiences. This improved regulation is proposed to culminate in a deepened capacity for meaning-making and greater life fulfillment (Garland et al., 2009). Mindfulness is, additionally, associated with improving compassion and kindness toward oneself and others (Neff, 2003). Skills of compassion and kindness seem to accordingly protect from self-criticism and rumination by improving emotional connection to others (*via* increasing empathy and reducing emotional reactivity; Van Doesum et al., 2013; Lin et al., 2016; Campos et al., 2019).

Studies investigating the potential benefits of mindfulness-based interventions to dementia caregivers highlight the importance of two fundamental pillars that mindfulness aims to cultivate: acceptance and a non-judgmental awareness (Spira et al., 2007; Jaffray et al., 2015; Krishnan et al., 2017). Adopting mindfulness skills has been shown to help caregivers acknowledge difficulties in their caregiving role and cultivate a focus on the present moment, rather than on past or future events. Mindfulness-based interventions can also reduce caregiver burden and distress, ultimately cultivating skillful coping strategies. For instance, caregivers of patients with HD, after undergoing mindfulness-based cognitive therapy, reported reduced psychological distress coupled with improved emotion regulation. Consequently, ruminative and worrying thoughts were reduced, highlighting that focusing on the present moment reduces distress and allows savoring of the present (Eccles et al., 2021).

Despite some studies showing evidence that mindfulness and compassion impact social cognition (e.g., empathy), very little is known about the association between mindfulness and relationship quality in dementia patient-caregiver dyads. It remains unclear whether mindfulness can improve relationship quality between caregivers and demented patients, making them more closely connected, empathic, or positive. However, by broadening caregivers’ perspective and helping them regulate their automatic reactions, mindfulness may support caregivers’ ability to respond more effectively to challenging communications in the context of dementia care (Garland et al., 2009; Lindsay and Creswell, 2017).

To further investigate those questions, we designed a randomized controlled trial to study the effectiveness of the novel mindfulness-based intervention we specifically tailored to the needs of dementia caregivers. We hypothesize that the mindfulness intervention, compared to the active control group, will lead to (A) an improved relational well-being (primary outcome), (B) a greater psychological well-being (secondary outcome), and (C) a positive impact on dementia patients’ activities (tertiary outcome).

## Methods and analysis

### Design

We will conduct a randomized, controlled trial in which participants will be randomly allocated to either the intervention group (an 8-week mindfulness course tailored to dementia caregivers’ needs), or to the active control arm mimicking the intervention group’s structure (an online self-guided 8-week emotion regulation program). Participants will undergo mixed-method assessments at three time points: baseline (T0, at month 0), post-intervention (T1, at month 2), and after the maintenance phase (T2, at month 5). The study is designed and powered to gather preliminary information on the intervention at the level of an exploratory pilot study. The intervention is designed to be appropriate for caregivers of patients with all categories of dementia, including those with primary deficits in memory, behavior, language, other cognition, or motor functioning.

### Setting

The study will be run virtually in the United States at the University of California, San Francisco (UCSF). Dementia caregivers will be recruited through a dementia clinic research registry, and by local advertisements and recruitment talks; caregivers do not need to be local to the San Francisco Bay Area to participate. The mindfulness-based intervention group will follow a live-online and interactive program (*via* Zoom). It will be led by two certified MBSR teachers who completed their advanced teacher training at the Centre for Mindfulness Studies (Toronto, Canada). The active control group will follow an online, self-guided emotion regulation program and will participate in support group discussions with other caregivers, held twice during the 8 weeks and moderated by a clinical psychologist (*via* Zoom).

### Participants

Eligible participants need to be (1) 18 years of age or older, (2) caregiving for a person with dementia in their personal life with regular (at minimum weekly) contact with them, (3) English speaking, (4) literate (i.e., being able to read course material), (5) able to attend weekly online classes *via* Zoom, and (6) willing to be randomized and participate in one of two interventions. Participants will be excluded from the study on the basis of (1) regularly practicing mindfulness meditation, mindful yoga, similar mindfulness activities or having received prior formal training in MBSR in the past 5 years, (2) currently experiencing active or unresolved trauma without professional psychological assistance, (3) diagnosis of psychosis (e.g., schizophrenia, schizoaffective disorder, or bipolar disorder according to the DSM-IV) or undergoing treatment for substance abuse, (4) acute suicide plans as measured by the Patient Safety Screener (PSS-3), (5) clinical diagnosis of dementia, and (6) uncorrected vision or hearing severe enough to limit their participation in the study.

### Sample size calculation

Because this is a novel study and there are no published effect sizes for the impact of this mindfulness-based intervention on our various outcomes, this study will establish preliminary effect sizes for all measures. Due to the highly interpersonal nature of the mindfulness intervention, enrolling more than 20 participants per arm was impractical; however, considering expected attrition, this sample size will only be powered to detect large between-group effect sizes (i.e., 80% power to detect *r* > =0.70; 25% power to detect *r* > = 0.50).

### Recruitment, consent, and allocation to interventions

Participants will be recruited by local advertisements and recruitment talks in the San Francisco Bay Area, online dementia caregiving groups, and by accessing an established research registry database. Recruitment is planned to continue until 40 eligible participants are enrolled (20 per intervention arm), and the approximate recruitment period is 2 months. Participants will not receive financial compensation for taking part in the study, and there will be no inducements for completing interventions. Participants deemed suitable for recruitment, will be contacted *via* email or phone with an opportunity to participate in additional eligibility screening, and to complete an online screening questionnaire to confirm eligibility. A follow-up email will include a review of study details (e.g., start date, expectations).

All screening and recruitment conversations will be completed by research personnel trained in the ethical conduct of human subjects’ research. Informed written consent will be obtained *via* interview with a trained examiner (*via* Zoom), during which the study will be clearly explained in detail, and caregivers will be able to ask questions prior to enrollment. Caregivers will also be informed that they can drop out of the study at any time. After this conversation, those agreeing to participate will be asked to formally consent to study participation. Due to current pandemic restrictions, DocuSign will be used to obtain participants’ consent signatures electronically. The amount of time expected for this consenting process is 20 min.

**Figure 1:**
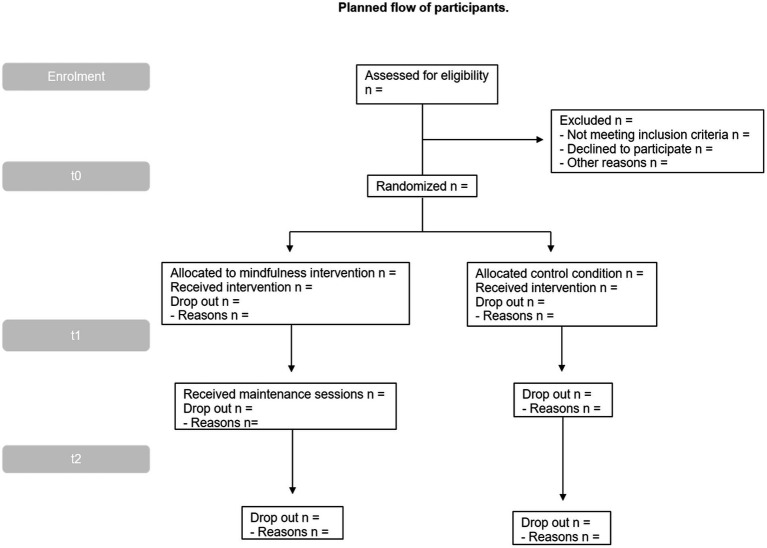
Planned flow of participants.

Next, for the baseline assessment part of the study (T0, 0 months), enrolled participants will be scheduled to participate in a 10-min semi-structured online interview *via* Zoom (see Supplementary material) and will complete a set of online surveys and performance tasks *via* Qualtrics. Those evaluations are designed to minimize possible subject fatigue, stress, and boredom. Breaks from testing will be allowed, and testing sessions may also be broken into multiple sessions. After all participants have completed enrollment and baseline assessment, they will be randomized to one of the two study arms and will then be informed *via* email of the group to which they have been assigned (see Figure 1; Table 1).

**Table 1:** Schedule of enrolment, interventions, and assessments.

Timepoint	Study period				
	Enrolment	Randomization	Intervention		Close-out
	*t_−1_*	*t_0_*	*t_1_*	*t_2_*	*t_x_*
Enrolment					
Eligibility screen	X				
Informed consent	X				
Allocation		X			
Interventions					
*MBSR adapted program*		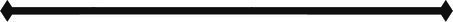			
*GARDEN*		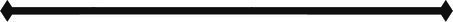			
Assessments					
*Primary outcome*		X	X	X	
*Secondary outcome*		X	X	X	
*Tertiary outcome*		X	X	X	

Randomization with 1:1 allocation ratio to one of the two arms will be conducted by a study investigator with no participant contact. Participants will be randomly allocated *via* a computerized random number sequence, generated *via* the R software (Team, 2014). Allocation will remain concealed from the study staff and participants during screening until randomization is completed.

### Interventions

#### Mindfulness-based intervention (interactive program)

Caregivers allocated to this experiential group will take part in an adapted mindfulness-based stress reduction (MBSR) program, formatted as a live class led by two certified MBSR instructors *via* Zoom, where they practice and receive feedback on mindfulness skills. To maintain group cohesiveness, while maximizing study enrollment, two separate classes of 10 caregivers each will be conducted concurrently. Recognizing that caregivers report having restricted time availability, this mindfulness-based intervention will have shortened sessions (1.5 h each) compared to the traditional MBSR 2.5 h-sessions, to better accommodate caregivers’ needs. MBSR adaptations with abbreviated weekly sessions and daily time commitment have been shown to still render significant decreases in perceived stress (Klatt et al., 2008; Carmody et al., 2009; Hoppes et al., 2012). Caregivers will be trained in meditation practices, learn about mindfulness, and stress theory, and have group discussions covering topics such as dementia caregiving challenges, and grief in dementia caregiving. The program curriculum (see Table 2) extends over a period of 8 weeks, where participants are encouraged to complete home practices and review reading material between live sessions.

**Table 2:** Weekly MBSR adapted course content.

	Topic	Content
Session 1	Introduction to mindfulness; Finding Stillness	Introducing caregivers to the notion of mindfulness as a way of being, exploring their needs and expectations from their participation, setting the group environment, and defining the term of “awareness” as a fundamental pillar of mindfulness
Session 2	Perception and its relation to stress; Perception and Perspective Taking	Highlighting the importance of commitment to practices, exploring the notion of a “non-judgmental” attitude toward the participants’ handling of practice as well as challenges, introducing the exploration of different angles of perception and perspective taking, and investigating the “habits & barriers” set by our automatic responses
Session 3	The power of being present; There is Pleasure & Power in Being Present	Expanding the awareness toward the present moment with a “no striving” attitude, introducing the concept of “power of choice” to caregivers and their challenges, deepening of mindfulness practices, and encouraging caregivers to expand on their awareness of noticing and appreciating pleasant moments in any experience
Session 4	The relation between mindfulness and stress; How Does Mindfulness Relate to Stress?	Exploring the relationship between stress reactivity, automaticity, and mindfulness, investigating maladaptive coping mechanisms stemming from stressful situations, modelling “acceptance” in caregivers’ experiences, expanding on the concept of grief in caregiving, and exploring the physiological territory of stress
Session 5	The difference between responding and reacting; Responding vs. Reacting	Introducing the concept of mindfully responding rather than automatically reacting to stress, considering the role of reactivity and its effects on health and suffering, discussing the role of feelings (e.g., anger, sadness, and grief), understanding the importance of being able to express feelings effectively, practicing acceptance, and assessing commitment and practicing in the middle of the intervention
Session 6	Learning about stressful communications; Stressful Communications	Shifting and extending focus from practicing intrapersonal to interpersonal mindfulness, learning to maintain inner balance while interacting, being aware of feelings, sensations, and thoughts while interacting with others, especially under conditions of acute or chronic stress, introducing the attitude of “letting-it-be & letting-it-go,” and exploring difficult communications in dementia caregiving
Retreat	Mindfulness Day/Silent Retreat	Three (3)-hour silent retreat. Extending mindfulness practices in a variety of approaches, reinforcing and deepening of mindfulness practices, while staying in silence for the duration of the retreat
Session 7	Learning and exploring self-resilience; Cultivating self-resilience	Expanding practices to daily caregiving life, introducing the fundamental mindfulness aspect of “trust” into daily life and caregivers’ challenges, cultivating psychological self-resilience, exploring the territory of self-compassion and self-reflection
Session 8	Continued practice, Wrap-up; Endings are New Beginnings	Reflecting on experiences of participations, cultivating the attitudes of “gratitude & generosity,” exploring the possibility of writing a personal letter for a recommitment to the individual practices in the future, overviewing of all practices, and unwrapping the dementia gifts for the mindful caregiver

The intervention is based on the manual of MBSR (Santorelli et al., 2020) and has been customized to the needs of dementia caregivers. To tailor the program, MBSR facilitators executed an in-depth literature search about challenges associated to dementia caregiving (Manteau-Rao and Barrows, 2016) and modified course material to explicitly integrate dementia caregiving concerns and experiences into the educational and practical material wherever relevant. Drawing from previously established mindfulness-based dementia care approaches, the focus of our mindfulness-based intervention is the development of mindfulness practice, sustaining attitudinal changes, reducing stress, and enhancing interactions throughout the caregiving journey (Dioquino et al., 2016). The structure of every session includes (1) educational input and scientific background to course elements, (2) mindfulness practices, (3) reflection of one’s practice and experience, (4) role plays or group exercises/discussions, and (5) home assignment discussions.

#### Active control group (self-guided program)

Caregivers allocated to this study arm will be assigned to a self-guided, online, weekly program that specifically targets positive emotion (Cheung et al., 2018; Addington et al., 2019; Moskowitz et al., 2019). The material aims to cultivate skills such as positive reappraisal, gratitude, self-compassion, and setting manageable goals. The program consists of skills introduced gradually over a 6-week period that caregivers will learn about and practice independently. Caregivers will also be asked to complete a daily questionnaire recording their feelings. To better mimic the structure of the active MBSR arm, which is in-person weekly, participants in this control arm will also be asked to participate in two online, interactive group discussions with other caregivers (*via* Zoom) at Week 4 and Week 8, facilitated by the study’s research director. This component will provide a space for caregivers to interact in person with others and discuss stressful experiences and application of skills learned to daily caregiving challenges. Study personnel are able to monitor the degree of completion of each module and will communicate with any caregiver who seems to be falling behind or otherwise not completing the work.

### Outcome measures

Assessment methods (see Table 3) will include standardized self-report questionnaires, task performance measures, and qualitative measures that will be collected at three timepoints (Months 0, 2, and 5). Additionally, at baseline, demographics will be collected for evaluation as potential confounds.

**Table 3:** Measurement description by domain.

	Type	Content	Category	Cronbach’s alpha	Items
EACQ	Explicit	Caregivers’ experiential avoidance	Relational well-being	0.60–0.71	15 items, 5-point Likert scale
ZBI-12	Explicit	Caregiver burden	Relational well-being	0.69–0.89	12 items, 5-point Likert scale
PAC	Explicit	Positive feelings from care provision	Relational well-being	0.80–0.86	9 items, 5-point Likert scale
IRI	Explicit	Empathic tendencies and feelings	Relational well-being	0.70–0.78	28 items, 5-point Likert scale
FCS	Explicit	Caregivers’ worry about compassion	Relational well-being	0.84–0.92	38 items, 5-point Likert scale
FCI-MS	Explicit	Perceived relationship quality	Relational well-being	>0.90	15 items, 5-point Likert scale
TASIT-SIE	Implicit	Assessing ability to detect sarcastic remarks and lies	Relational well-being	–	17 items, yes/no
HUMOR	Implicit	Assessing humor comprehension	Relational well-being	–	–
SDR	Implicit	Knowledge of social norms around emotional displays	Relational well-being	–	–
DASS-21	Explicit	Depression, anxiety, stress	Psychological well-being	0.86–0.90	21 items, 4-point Likert scale
DERS	Explicit	Trait-level emotion regulation ability	Psychological well-being	0.80–0.89	36 items, 5-point Likert scale
SPANE	Explicit	Positive & negative feelings associated to an experience	Psychological well-being	0.81–0.89	12 items, 5-point Likert scale
SES	Explicit	Perceived self-efficacy	Psychological well-being	–	4 items, 5-point Likert scale
FFMQ-SF	Explicit	Perceived levels of trait mindfulness	Psychological well-being	0.75–0.93	15 items, 5-point Likert scale
SCS-SF	Explicit	Caregivers’ self-compassion	Psychological well-being	>0.86	12 items, 5-point Likert scale
HEMA-R	Explicit	Psychological wellbeing	Psychological well-being	>0.80	10 items, 7-point Likert scale
WHOQOL-BREF	Explicit	Quality of life improvements	Psychological well-being	0.83	Domains, 5-point Likert scale
PES-AD	Explicit	Frequency of events & pleasantness of events	Dementia Patient Lifestyle	0.86–0.95	40 items, 3-point Likert scale

#### Primary outcome: Relational well-being

We hypothesize that participants in the MBSR intervention will experience positive changes in their interpersonal relationships and their ability to perceive and correctly interpret social signals from pre- to post-intervention, and that these changes will be more pronounced than those seen in the active control group. We included a number of measures of caregivers’ relational well-being.

To measure caregivers’ experiential avoidance, the Experiential Avoidance Caregiver Questionnaire (EACQ) will be used (Losada et al., 2014). Participants will rate their tendency to avoid unwanted caregiving experiences on a 5-point Likert scale from 1 (not at all) to 5 (a lot). The EACQ has been shown to have good internal consistency (Cronbach’s alpha; 0.60–0.71). The validity indexes suggest that EACQ is associated with other scales or constructs (e.g., anxiety, depression or alexithymia) in the expected directions. The EACQ may prove to be a useful tool for the identification of avoidance which can be frequently observed in distressed dementia caregivers, and which may explain the maintenance or exacerbation of their high levels of anxiety or stress.

To examine subjective sense of caregiver burden, the Zarit Burden Interview short form (ZBI-12), will be used (Higginson et al., 2010). Participants will rate their perceived burden on a 5-point Likert scale from 0 (*never*) to 4 (*nearly always*). The ZBI-12 has been shown to have good internal consistency (Cronbach’s alpha; 0.69–0.89). No loss of reliability or validity appear to result from reduction of items from the original scale. Consistent with the findings previously reported this measure appears to be an effective measure of caregiver burden (Bédard et al., 2001).

The Positive Aspect of Caregiving (PAC) scale will be used to assess positive feelings resulting from care provision among family caregivers of older adults with functional limitations (Siow et al., 2017). The 9-item PAC scale is a 5-point Likert scale, where participants will rate their positive aspects of caregiving from 1 (disagree a lot) to 5 (agree a lot). Three resulting scores can be derived and used independently in analyses: overall PAC score, SA subscale score (Self-Affirmation), and OL (Outlook-on-Life) subscale. The PAC has been shown to have good internal consistency (Cronbach’s alpha; 0.80–0.86) and is considered a reliable and valid measure for assessing the positive benefits gained by the family caregivers when providing care.

The Interpersonal Reactivity Index (IRI) will be used for assessing caregivers’ empathic functioning (Davis et al., 1980). The IRI yields four subscales of empathy (PT; Perspective Taking-cognitive, FS; Fantasy-cognitive, PD; Personal Distress, EC; Empathic Concern). Participants will rate their empathic tendencies and feelings on a 5-point Likert scale from “*Does not describe me well*” to “*Describes me very well*.” The IRI has been shown to have good internal consistency (Cronbach’s alpha; 0.70–0.78). The IRI subscales will measure caregiver’s empathy as various types of both cognitive processes (perspective taking and fantasy subscales) and affective processes (empathic concern and personal distress subscales).

We will use the Fears of Compassion Scale (FCS), to evaluate caregivers’ worry about compassion to others and themselves (Gilbert, 2014). The FCS yields three subscales (expressing compassion for others, responding to compassion from others, expressing kindness and compassion toward yourself). Participants will rate their worry on a 5-point Likert scale from 0 (*do not agree at all*) to 4 (*completely agree*). FCS has been shown to have good internal consistency (Cronbach’s alpha; 0.84–0.92) and encompasses important implications for therapeutic interventions because affiliative emotions are major regulators of worry-based emotions.

We will use the Mutuality Scale of the Family Care Inventory (FCI-MS), to assess mutuality in relationship between the caregiver and person with dementia, from the caregiver’s perspective (Pucciarelli et al., 2016). Caregivers will rate their perceived relationship quality with their care-recipient on a 5-point Likert scale from 0 (*not at all*) to 4 (*a great deal*). The FCI-MS has been shown to have good internal consistency (Cronbach’s alpha; above 0.90) and is considered a valid and reliable instrument to measure mutuality between patients and caregivers. We will also include direct tests of caregivers’ socioemotional sensitivity and interpersonal cue processing. The capacity to read and correctly interpret contextualized social signals will be assessed *via* The Awareness of Social Inference Test-Social Inference Enriched (TASIT-SIE). TASIT-SIE comprises of conversational exchanges with vignettes containing insincere communication (Honan et al., 2016) and yields multiple scoring subscales on exchanges that involve lying and sarcasm (i.e., doing, saying, thinking, feeling).

The Humor test, developed and validated at UCSF with neurologically healthy older adults, will be used to evaluate humor comprehension. Participants will read predominantly non-verbal cartoon strips missing the last frame, and then select one of four optional endings depicting correct funny (*CF*), straightforward (SF), humorous nonsequitur (HNS), and unrelated nonsequitur (UNS) conclusions. Final scores describe whether participants are sensitive to the “logic” and “surprise” elements of humor.

The Social Display Rules task (SDR), also developed and used at UCSF, will be used to assess caregivers’ knowledge of social norms around emotional displays. Participants will receive descriptions of various social scenarios, and then be asked to imagine the socially appropriate emotional response to them. In each scenario, the socially appropriate response involves altering the immediate emotional reaction. Participants will be asked to select the picture of an emotional face reflecting how they “should” look during the scenario. Subscale scores will be obtained for minimization, intensification, and substitution of an emotion, and a control subscale score will be obtained for selecting the correct emotion when no alteration is socially required.

As part of relational wellbeing, *emotion*, *connectedness*, and *rigidity* statements between caregivers and their care-recipients will be assessed *via* an audio-recorded 10-min semi-structured interview. Verbatim transcripts will be prepared and analyzed using a text analysis software Oedipus Text (Ascher et al., 2010; Connelly et al., 2020). We will assess socioemotional language usage in caregivers across three domains (see Supplementary material):

- *Emotion*: Consistent with other studies of emotional language usage (positive and negative), a composite list consisting of words from the emotional lexicon state will be used.
- *Connectedness:* We will identify the number of pronouns in each of the following three lexical categories, as defined by the pronoun lexicon: we-words (pronouns referring to the couple), me-words (pronouns referring to the speaker) and you-words (pronouns referring to the other spouse).
- *Rigidity*: We will identify the number of rigid statements (e.g., never) regarding the care recipient’s behavior or characteristics, as defined by the rigidity lexicon.

After initial independent scoring, a consensus review will be performed to confirm reliability and validity of scoring for all analyses. For each domain, mean composites will be generated. In addition, exploratory qualitative analysis will be performed to examine themes in all three domains.

#### Secondary outcome: Psychological well-being

We hypothesize that the mindfulness-based intervention will have positive effects on participants’ psychological functioning, including their mental health, emotion regulation, and quality of life.

To examine this domain, we will measure overall mental health using the Depression, Anxiety and Stress Scale-21 (DASS-21; Gloster et al., 2008). Participants will rate statements and their application to their own experience during their past week, generating three subscales (depression, anxiety and stress respectively). The DASS-21 has been shown to have good internal consistency (Cronbach’s alpha; 0.86–0.90) and its depression and anxiety subscales show good correlations with self-rating depression scale and state trait anxiety inventory.

The Difficulties in Emotion Regulation (DERS) scale will be used to assess each participant’s trait-level emotion regulation ability (Gratz and Roemer, 2004). The DERS yields six subscales: (a) lack of emotional awareness (*Awareness*; “I am attentive to my feelings,” reverse-scored); (b) lack of emotional clarity (*Clarity*; “I have difficulty making sense out of my feelings”); (c) difficulty regulating behavior when distressed (*Impulse*; “When I’m upset, I become out of control”); (d) difficulty engaging in goal-directed cognition and behavior when distressed (*Goals*; “When I’m upset, I have difficulty getting work done”); (e) unwillingness to accept certain emotional responses (*Non-acceptance*; “When I’m upset, I become angry at myself for feeling that way); and (f) lack of access to strategies for feeling better when distressed (*Strategies*; “When I’m upset, I believe there is nothing I can do to feel better”). Overall, the DERS has high internal consistency (Cronbach’s alpha; 0.80–0.89), good test–retest reliability, and predictive validity. However, a wide array of follow up studies (Hallion et al., 2018) highlight that the Awareness subscale seem to show relatively poor psychometric properties, concluding that the DERS is psychometrically stronger when the *Awareness* subscale is excluded.

We will use the Scale of Positive and Negative Experiences (SPANE), to assess participants feelings’ valence (positive or negative) toward experience (Diener et al., 2009). The SPANE gives three scores: (a) summed positive (SPANE-P), (b) negative scale (SPANE-N) score, and (c) the two scores combined by subtracting the negative from the positive score, and the resulting SPANE-B scores. SPANE-B shows balance between positive and negative scores. The SPANE has good reliability and convergent validity with other measures of emotion, well-being, happiness, and life satisfaction. The three subscales have high internal consistency (Cronbach’s alpha; 0.81–0.89).

To measure caregivers’ self-efficacy (Merrilees et al., 2020), we will use the Care Ecosystem Self Efficacy Scale (SES).

Characteristics of mindfulness will be assessed *via* the Five Facet Mindfulness Questionnaire-Short Form (FFMQ-SF), yielding five subscales (Observing, Describing, Acting with awareness, Nonjudging, Reactivity). Participants will rate phrases according to their perceived mindfulness (Bohlmeijer et al., 2011). The FFMQ-SF accurately identifies varying levels of trait mindfulness and has been shown to have good internal consistency (Cronbach’s alpha; 0.75–0.93). Importantly, studies utilizing this instrument report increases in individual’s FFMQ scores with participation in MBSR programs (Baer et al., 2012).

The Self-Compassion Scale-Short Form (SCS-SF) will be used to assess caregivers’ self-compassion (Neff, 2003). The SCS-SF yields six subscales (Self-Kindness, Self-Judgment, Common Humanity Items, Isolation, Mindfulness, Over-identification). The SCS-SF has been shown to have good internal consistency (Cronbach’s alpha; above 0.86). Research has shown that self-compassion is associated with psychological well-being and is an important protective factor that fosters emotional resilience, with higher levels of self-compassion to be typically related to greater psychological health as demonstrated by less depression and anxiety (Raes, 2011).

Psychological well-being will be measured *via* the Hedonic and Eudaimonic Motives for Activities-Revised (HEMA-R) scale (Huta and Ryan, 2009). The HEMA-R yields two scoring subscales: 1. hedonic; personal importance of pleasure and absence of pain and 2. eudaimonic; personal importance of authenticity, excellence, and growth. Participants will rate to what extent they experience their lives as valuable. The HEMA-R has been shown to cohere as a single distinct factor and has good internal consistency (Cronbach’s alpha; above 0.80). Eudaimonic and hedonic motives exhibit beneficial effects on a wide range of positive outcomes such as emotion regulation, coping strategies and well-being (Ortner et al., 2018).

An additional measure pertaining to psychological well-being will be the WHO-quality of life (WHOQOL-BREF) questionnaire. The WHOQOL-BREF will be used to assess quality of life improvements in domains of physical health, psychological, social relationships, and environment (THE WHOQOL GROUP, 1998). The WHOQOL-BREF has been shown to have good internal consistency (Cronbach’s alpha; 0.83) and can be used to measure caregiver’s subjective experiences of their own physical, mental and social well-being.

#### Tertiary outcome: Dementia patient lifestyle/activities

While we predict that direct effects on the caregiver will be the most likely primary and secondary outcomes, we also wish to evaluate the degree to which any changes impact the lives of dementia patients themselves. Because we are not directly enrolling or evaluating patients in the study and will not compromise their confidentiality by identifying them in any way, we will gather indirect evidence of activity changes through caregivers’ subjective reports.

Lifestyle changes in the person with dementia will be measured by asking caregivers to complete the Pleasant Event Schedule-AD (PES-AD). The PES-AD yields two subscales (Frequency of events, Pleasantness of events), and assess the frequency and pleasure the person with dementia receives from daily activities (Logsdon and Teri, 1997). The PES-AD has been shown to have good internal consistency (Cronbach’s alpha; 0.86–0.95).

### Data protection

The institution has asserted that a Data Monitoring Committee will not be needed because both interventions convey a low potential for harm. We will, nonetheless, have an internal study group that will be monitoring participants’ well-being (i.e., graduate students, psychologists, formal graduate student of psychology), and will report back to the institutional report board if any adverse events arise. Privacy information and contact preference will be stored in private internal research UCSF database. We will use a unique identification code for each participant and all coded data will be maintained in computers equipped with security programs. We will not use individual identities in any reports or publications without expressed written permission and no data will be added to medical/clinical records. The active control group will be assignment of a new, non-identifiable email account (key linking private email address) which will be only available to UCSF research team. Participants’ responses to written material will be private and recordings of participants’ interviews will be stored on a HIPAA-secure research server until transcribed, saved under deidentified file names, and be accessible only by approved research personnel (for transcription). All recording copies will be destroyed after transcriptions are performed/when the study is completed.

### Data analysis plan

The study will use mixed methods, integrating quantitative and qualitative strategies, making no assumptions about linear relationships between quantitative and qualitative data/findings (DePoy and Gitlin, 1994). These two strategies will be employed as different ways of exploring and understanding the theme in question, i.e., mindfulness and caregiver wellbeing.

Regarding quantitative measures we will first identify factors that are confounders between the two intervention groups, which may include socio-demographics, relationship to care recipients, healthcare access, socioemotional support, dementia patient diagnosis, engagement in intervention, relationship quality with facilitator, hours (weekly) spent with the person with dementia. Any variables showing significant group differences will then be considered confounds and included in the main statistical analyses. We will also check for missing data and signs of systematic response bias in self-reported responses. The main analysis will evaluate the effect of treatment group x time (t0, t1, t2). All participant outcome measures (domains: relational well-being, psychological well-being, dementia patient’s lifestyle changes), will be analyzed using generalized linear mixed models. Models will treat treatment and time points (including their interaction) as fixed effects and caregivers as random effects. We will look for a main treatment effect, a main time effect, and an interaction effect of treatment by time. All hypotheses will be individually evaluated at a nominal *p*-value of 0.05.

Semi-structured interviews will be analyzed using thematic analysis. First, grounded theory’s open coding stage will be employed, followed by axial coding and analyses (Spradley, 1979; Charmaz, 2006). We will identify meaningful themes and conceptualize our findings in relation to existing mindfulness theories and stress reactivity research. Because the first two authors are mindfulness teachers who have worked extensively with older adults, including dementia caregivers, qualitative data will be analyzed separately and jointly by the first two authors. Final synthesis of analyzed data will be obtained through continuous discussions among the research team until consensus is reached.

### Fidelity of implementation

Quality of the mindfulness-based intervention will be ensured through the engagement of two certified MBSR instructors with experience in practicing and teaching mindfulness *via* Zoom. These two instructors will co-lead each session and will undergo weekly debriefing with the study lead (a clinical psychologist) and their MBSR supervisor to resolve any implementation issues.

### Strategies for improving adherence

Throughout the study, participation in both arms will be monitored, and retention enhanced *via* emails and calls. In the mindfulness-based intervention participants attending at least five of eight sessions will be defined as completers and will be included in the final analysis, though the number of completed sessions will be evaluated as a potential confound. To document participants’ daily mindfulness practice throughout the course, a weekly online survey will be employed accounting for home practice frequency and quality. In the active control arm, participants’ progress and skill completion can be monitored online by study staff; participants completing 75% of the material before the T1 assessment and attending at least one in-person session will be included in the final analysis, though different levels of completion will be evaluated as a potential confound.

Interventions may be discontinued at the participant’s request, with case-by-case evaluation of causative factors (e.g., death of care-recipient, acute trauma, intense amount of stress/grief, scheduling conflict). Any participant not meeting the preset participation thresholds will not be excluded from completing the remainder of the study, but their data will not be analyzed. Some attrition is expected, but with enhanced retention *via* direct contact with participants, we believe we can retain 80% of participants through the entire intervention and T1 assessment.

While we suggest a fairly broad array of assessment measures in this protocol, the overall time to complete them is ~1 h. In our two decades of experience performing observational research with caregivers of dementia patients, we have found that 1 h of questionnaires is not typically experienced as unduly burdensome for these individuals. However, individuals with more extreme current stressors could find the time demand for participation in the protocol daunting, thus they are fully informed of the demands on their effort during the informed consent process, at which time they will have the option to decline to participate.

### Procedures to reduce bias

Participants will be randomly allocated with 1:1 allocation ratio to one of the two arms *via* a computerized random number, generated *via* the R software (Team, 2014). The numeric random sequence document will be unavailable to the researcher in charge of enrolling and assessing participants, to avoid research bias. Enrollment interviews, consenting, and assignment of group allocation will be performed by research team members other than those who will facilitate the mindfulness-based class or the caregiver support group. Participants and group facilitators cannot be blind to arm allocation due to each intervention’s nature (distinct, identifiable material and procedures). Investigators and instructors have undergone formal training in the reduction of cultural bias in research settings.

## Discussion

This RCT protocol is designed to provide valuable information about the manner in which dementia caregivers’ well-being is improved by mindfulness-based interventions that are tailored to their specific lifestyle and stressors. Developing a capacity to broaden perspective without automatically reacting, a core mindfulness skill, may make the perceived relationship quality between caregiver and demented patient seem more mutual, connected, empathic and positive, despite the ongoing stressors of dementia care. Caregivers of persons with dementia receiving the 8-week mindfulness intervention may also gain a new perspective on their role as a caregiver, potentially resulting in increased positive affect and reframe their caregiving experience as more purposeful. Overall, this study will contribute to the evidence base concerning whether mindfulness-based interventions can be efficacious in empowering caregivers to regain relationship satisfaction and achieve greater equanimity in the face of their substantial stressors. In addition, this study will inform future studies with larger samples.

The design of the current protocol is subject to limitations. For instance, a small group size is necessary for the active intervention, leading to an overall small sample size, a selective recruitment pool, and an overall homogeneous sample. It is a risk that, if only a single caregiver group is run through the active intervention, the study might then be underpowered to definitively establish the effectiveness of the proposed intervention. However, additional rounds of intervention may increase the overall sample size to a more acceptable level of statistical power. While longer follow-up periods are very informative and important, the scope of our study is to measure short term effects of mindfulness-based interventions on caregiver wellbeing. Hence a 3 month follow up fit better our pilot study’s scope. Also, the study is designed to provide both qualitative responses and quantitative trends that will add to the existing evidence concerning the efficacy of mindfulness-based interventions for dementia caregivers. In addition, any social intervention it is contextualized to the dominant culture in which it was developed. Thus, this protocol may not generalize to other cultural and linguistic groups, and therefore a thoughtful and data-driven process should be used when adapting it more broadly to such groups.

Even with these caveats, however, we believe this study represents an important step in a much-needed line of investigation. Adaptation of existing, effective interventions for use with caregivers is a novel but important healthcare intervention. Evidence suggests that caregivers of persons with dementia are experiencing long-term, intensive stress (Connell et al., 2001; Sörensen and Conwell, 2011; George et al., 2020). Because mindfulness-based interventions are based in the foundational features of nonjudgmental awareness and acceptance of negative emotions and circumstances, they have untapped potential to positively impact the wellbeing of dementia caregivers (Jaffray et al., 2015).

## Ethics statement

This study was approved by the Institutional Review Board of the University of California, San Francisco (Reference number: 20-31240). The trial was registered at the United States National Library of Medicine Clinical Trials (ClinicalTrials.gov Identifier: NCT04977245). Potential changes to the study protocol will be reported to the ethics committee and will be updated in the trial registry.

## Author contributions

RA: conceptualization, writing–original draft preparation, resources, methodology, and writing–review and editing. DT: conceptualization, visualization, writing-original draft preparation, resources, and writing–review and editing. HL: conceptualization, methodology, investigation, resources, and writing–review and editing. PC: software, investigation, resources, data curation, and writing–review and editing. RC: investigation, data curation, resources, and writing–review and editing. BO: data curation, investigation, and writing–review and editing. AS: methodology, resources and writing-review and editing. SS: methodology, resources, and writing–review and editing. RL: methodology, software, resources, and writing–review and editing. NF: resources, validation, and writing–review and editing. JM: resources and writing–review and editing. KR: conceptualization, resources, supervision, project administration, and writing–review and editing. All authors contributed to the article and approved the submitted version.

## Funding

The planned study has no sponsor and was conducted via discretionary funding. AB’s time was supported by NIA K01AG059840.

## Conflict of interest

The authors declare that the research was conducted in the absence of any commercial or financial relationships that could be construed as a potential conflict of interest.

## Publisher’s note

All claims expressed in this article are solely those of the authors and do not necessarily represent those of their affiliated organizations, or those of the publisher, the editors and the reviewers. Any product that may be evaluated in this article, or claim that may be made by its manufacturer, is not guaranteed or endorsed by the publisher.

## References

[ref1] Addington E. L. Cheung E. O. Bassett S. M. Kwok I. Schuette S. A. Shiu E. . (2019). The MARIGOLD study: feasibility and enhancement of an online intervention to improve emotion regulation in people with elevated depressive symptoms. J. Affect. Disord. 257, 257, 352–364. doi: 10.1016/j.jad.2019.07.049, PMID: 31302525 PMC6711819

[ref2] Aldao A. Nolen-Hoeksema S. Schweizer S. (2010). Emotion-regulation strategies across psychopathology: a meta-analytic review. Clin. Psychol. Rev. 30, 217–237. doi: 10.1016/j.cpr.2009.11.004, PMID: 20015584

[ref3] Ascher E. A. Sturm V. E. Seider B. H. Holley S. R. Miller B. L. Levenson R. W. (2010). Relationship satisfaction and emotional language in frontotemporal dementia and Alzheimer disease patients and spousal caregivers. Alzheimer Dis. Assoc. Disord. 24, 49–55. doi: 10.1097/wad.0b013e3181bd66a3, PMID: 20220322 PMC2838197

[ref4] Aubeeluck A. V. Buchanan H. Stupple E. J. (2011). ‘All the burden on all the carers’: exploring quality of life with family caregivers of Huntington’s disease patients. Qual. Life Res. 21, 1425–1435. doi: 10.1007/s11136-011-0062-x, PMID: 22081218

[ref5] Baer R. A. Carmody J. Hunsinger M. (2012). Weekly change in mindfulness and perceived stress in a mindfulness-based stress reduction program. J. Clin. Psychol. 68, 755–765. doi: 10.1002/jclp.21865, PMID: 22623334

[ref6] Bédard M. Molloy D. W. Squire L. Dubois S. Lever J. A. O’Donnell M. (2001). The Zarit burden interview. Gerontologist 41, 652–657. doi: 10.1093/geront/41.5.652 11574710

[ref7] Bertrand R. M. Fredman L. Saczynski J. (2006). Are all caregivers created equal? Stress in caregivers to adults with and without dementia. J. Aging Health 18, 534–551. doi: 10.1177/0898264306289620, PMID: 16835388

[ref8] Bledin K. D. Maccarthy B. Kuipers L. Woods R. T. (1990). Daughters of people with dementia expressed emotion, strain and coping. Br. J. Psychiatry 157, 221–227. doi: 10.1192/bjp.157.2.221, PMID: 2224372

[ref9] Bohlmeijer E. ten Klooster P. M. Fledderus M. Veehof M. Baer R. (2011). Psychometric properties of the five facet mindfulness questionnaire in depressed adults and development of a short form. Assessment 18, 308–320. doi: 10.1177/1073191111408231, PMID: 21586480

[ref10] Boutoleau-Bretonnière C. Vercelletto M. Volteau C. Renou P. Lamy E. (2008). Zarit burden inventory and activities of daily living in the behavioral variant of frontotemporal dementia. Dement. Geriatr. Cogn. Disord. 25, 272–277. doi: 10.1159/000117394, PMID: 18285675

[ref11] Campos D. Modrego-Alarcón M. López-del-Hoyo Y. González-Panzano M. Van Gordon W. Shonin E. . (2019). Exploring the role of meditation and dispositional mindfulness on social cognition domains: a controlled study. Front. Psychol. 10:809. doi: 10.3389/fpsyg.2019.00809, PMID: 31031678 PMC6470267

[ref12] Carmody J. Baer R. A. Lykins L. B. Olendzki N. E. (2009). An empirical study of the mechanisms of mindfulness in a mindfulness-based stress reduction program. J. Clin. Psychol. 65, 613–626. doi: 10.1002/jclp.20579, PMID: 19267330

[ref13] Charmaz K. (2006). Constructing Grounded Theory: A Practical Guide Through Qualitative Analysis. London: Sage.

[ref14] Cheung E. O. Addington E. L. Bassett S. M. Schuette S. A. Shiu E. W. Cohn M. A. . (2018). A self-paced, web-based, positive emotion skills intervention for reducing symptoms of depression: protocol for development and pilot testing of MARIGOLD. JMIR Res. Protoc. 7:e10494. doi: 10.2196/10494, PMID: 29871853 PMC6008514

[ref15] Collins R. N. Kishita N. (2018). The effectiveness of mindfulness- and acceptance-based interventions for informal caregivers of people with dementia: a meta-analysis. Gerontologist 59, e363–e379. doi: 10.1093/geront/gny024, PMID: 29635303

[ref16] Connell C. M. Janevic M. R. Gallant M. P. (2001). The costs of caring: impact of dementia on family caregivers. J. Geriatr. Psychiatry Neurol. 14, 179–187. doi: 10.1177/089198870101400403 11794446

[ref17] Connelly D. E. Verstaen A. Brown C. L. Lwi S. J. Levenson R. W. (2020). Pronoun use during patient-caregiver interactions: associations with caregiver well-being. Dement. Geriatr. Cogn. Disord. 49, 202–209. doi: 10.1159/000508095, PMID: 32610328 PMC7805608

[ref18] Cooke D. D. McNally L. Mulligan K. T. Harrison M. J. Newman S. P. (2001). Psychosocial interventions for caregivers of people with dementia: a systematic review. Aging Mental Health 5, 120–135. doi: 10.1080/713650019 11511059

[ref19] Cooper C. Blanchard M. Selwood A. Walker Z. Livingston G. (2010a). Family carers’ distress and abusive behaviour: longitudinal study. Br. J. Psychiatry 196, 480–485. doi: 10.1192/bjp.bp.109.071811, PMID: 20513860

[ref20] Cooper C. Selwood A. Blanchard M. Walker Z. Blizard R. Livingston G. (2010b). The determinants of family carers’ abusive behaviour to people with dementia: results of the CARD study. J. Affect. Disord. 121, 136–142. doi: 10.1016/j.jad.2009.05.001, PMID: 19446884

[ref21] Dam A. E. H. de Vugt M. E. Klinkenberg I. P. M. Verhey F. R. J. van Boxtel M. P. J. (2016). A systematic review of social support interventions for caregivers of people with dementia: are they doing what they promise? Maturitas 85, 117–130. doi: 10.1016/j.maturitas.2015.12.008, PMID: 26857890

[ref22] Davis M. H. Davis M. P. Davis M. Davis M. Davis M. Davis M. (1980). A Multidimensional Approach to Individual Differences in Empathy. undefined. [online] Available at: https://www.semanticscholar.org/paper/A-Multidimensional-Approach-to-Individual-in-Davis-Davis/c717eb4e913c3249eac18d0fba13a1aa02d60dad.

[ref23] de Vugt M. E. Riedijk S. R. Aalten P. Tibben A. van Swieten J. C. Verhey F. R. J. (2006). Impact of behavioural problems on spousal caregivers: a comparison between Alzheimer’s disease and frontotemporal dementia. Dement. Geriatr. Cogn. Disord. 22, 35–41. doi: 10.1159/000093102, PMID: 16679763

[ref24] DePoy E. Gitlin L. N. (1994). Introduction to Research: Multiple Strategies for Health and Human Services, St. Louis: Mosby.

[ref25] Diener E. Wirtz D. Tov W. Kim-Prieto C. Choi D. Oishi S. . (2009). New well-being measures: short scales to assess flourishing and positive and negative feelings. Social Indic. Res. 97, 143–156. doi: 10.1007/s11205-009-9493-y

[ref26] Dioquino Y. W. J. Manteau-Rao M. Peterson K. Madison C. A. (2016). P1-445: preliminary findings from a study of mindfulness-based dementia care (MBDC) training: a method to enhance dementia caregiver well-being. Alzheimers Dement. 12:P605. doi: 10.1016/j.jalz.2016.06.1197

[ref27] Eccles F. J. R. Craufurd D. Smith A. Davies R. Glenny K. Homberger M. . (2021). Experiences of mindfulness-based cognitive therapy for Premanifest Huntington’s disease. J. Huntington’s Dis. 10, 277–291. doi: 10.3233/jhd-210471, PMID: 33646170

[ref28] Fearon M. Donaldson C. Burns A. Tarrier N. (1998). Intimacy as a determinant of expressed emotion in carers of people with Alzheimer’s disease. Psychol. Med. 28, 1085–1090. doi: 10.1017/s0033291798007156, PMID: 9794015

[ref29] Fontaine J. Jutlla K. Read K. Brooker D. Evans S. Dr A. (2016). The Experiences, Needs and Outcomes for Carers of People with Dementia Literature Review. [online] Available at: https://dora.dmu.ac.uk/bitstream/handle/2086/14058/RSAS%20Worcester%20literature%20review_08.04.16.pdf?sequence=1&isAllowed=y (Accessed September 30, 2022).

[ref30] Garland E. Gaylord S. Park J. (2009). The role of mindfulness in positive reappraisal. EXPLORE 5, 37–44. doi: 10.1016/j.explore.2008.10.001, PMID: 19114262 PMC2719560

[ref31] George C. Ferreira N. Evans R. Honeyman V. (2020). A systematic review of the association between individual behavioural and psychological symptoms in dementia and carer burden. Working Older People 24, 181–203. doi: 10.1108/wwop-06-2020-0024

[ref32] Gilbert P. (2014). The origins and nature of compassion focused therapy. Br. J. Clin. Psychol. 53, 6–41. doi: 10.1111/bjc.12043, PMID: 24588760

[ref33] Gloster A. T. Rhoades H. M. Novy D. Klotsche J. Senior A. Kunik M. . (2008). Psychometric properties of the depression anxiety and stress Scale-21 in older primary care patients. J. Affective Disord. 110, 248–259. doi: 10.1016/j.jad.2008.01.023, PMID: 18304648 PMC2709995

[ref34] Gratz K. L. Roemer L. (2004). Multidimensional assessment of emotion regulation and dysregulation: development, factor structure, and initial validation of the difficulties in emotion regulation scale. J. Psychopathol. Behav. Assess. 26, 41–54. doi: 10.1023/b:joba.0000007455.08539.94

[ref35] Guendelman S. Medeiros S. Rampes H. (2017). Mindfulness and emotion regulation: insights from neurobiological, psychological, and clinical studies. Front. Psychol. 8, 8:220. doi: 10.3389/fpsyg.2017.00220, PMID: 28321194 PMC5337506

[ref36] Hallion L. S. Steinman S. A. Tolin D. F. Diefenbach G. J. (2018). Psychometric properties of the difficulties in emotion regulation scale (DERS) and its short forms in adults with emotional disorders. Front. Psychol. 9:539. doi: 10.3389/fpsyg.2018.00539, PMID: 29725312 PMC5917244

[ref37] Hartelius L. (2010). Communication and Huntington’s disease: qualitative interviews and focus groups with persons with Huntington’s disease, family members, and carers. Int. J. Lang. Commun. Disord. 45, 381–393. doi: 10.3109/13682820903105145, PMID: 20144006

[ref38] Higginson I. J. Gao W. Jackson D. Murray J. Harding R. (2010). Short-form Zarit caregiver burden interviews were valid in advanced conditions. J. Clin. Epidemiol. 63, 535–542. doi: 10.1016/j.jclinepi.2009.06.014, PMID: 19836205

[ref39] Honan C. A. McDonald S. Sufani C. Hine D. W. Kumfor F. (2016). The awareness of social inference test: development of a shortened version for use in adults with acquired brain injury. Clin. Neuropsychol. 30, 243–264. doi: 10.1080/13854046.2015.1136691, PMID: 26918817

[ref40] Hoppes S. Bryce H. Hellman C. Finlay E. (2012). The effects of brief mindfulness training on caregivers’ well-being. Act. Adapt. Aging 36, 147–166. doi: 10.1080/01924788.2012.673154

[ref41] Huta V. Ryan R. M. (2009). Pursuing pleasure or virtue: the differential and overlapping well-being benefits of hedonic and Eudaimonic motives. J. Happiness Stud. 11, 735–762. doi: 10.1007/s10902-009-9171-4

[ref42] Jaffray L. Bridgman H. Stephens M. Skinner T. (2015). Evaluating the effects of mindfulness-based interventions for informal palliative caregivers: a systematic literature review. Palliat. Med. 30, 117–131. doi: 10.1177/0269216315600331, PMID: 26281853

[ref43] Kabat-Zinn J. (1992). Review of full catastrophe living: using the wisdom of your body and mind to face stress, pain, and illness. Contemp. Psychol.: J. Rev. 37:609. doi: 10.1037/032287

[ref44] Killingsworth M. A. Gilbert D. T. (2010). A wandering mind is an unhappy mind. Science 330:932. doi: 10.1126/science.1192439, PMID: 21071660

[ref45] Klatt M. D. Buckworth J. Malarkey W. B. (2008). Effects of low-dose mindfulness-based stress reduction (MBSR-ld) on working adults. Health Educ. Behav. 36, 601–614. doi: 10.1177/1090198108317627, PMID: 18469160

[ref46] Krishnan S. York M. K. Backus D. Heyn P. C. (2017). Coping with caregiver burnout when caring for a person with neurodegenerative disease: a guide for caregivers. Arch. Phys. Med. Rehabil. 98, 805–807. doi: 10.1016/j.apmr.2016.11.002, PMID: 28185639

[ref47] Lea Steadman P. Tremont G. Duncan Davis J. (2007). Premorbid relationship satisfaction and caregiver burden in dementia caregivers. J. Geriatr. Psychiatry Neurol. 20, 115–119. doi: 10.1177/0891988706298624, PMID: 17548782 PMC1890033

[ref48] Leggett A. N. Zarit S. Taylor A. Galvin J. E. (2011). Stress and burden among caregivers of patients with Lewy body dementia. Gerontologist 51, 76–85. doi: 10.1093/geront/gnq055, PMID: 20667944

[ref49] Li G. Yuan H. Zhang W. (2016). The effects of mindfulness-based stress reduction for family caregivers: systematic review. Arch. Psychiatr. Nurs. 30, 292–299. doi: 10.1016/j.apnu.2015.08.014, PMID: 26992885

[ref50] Lin Y. Fisher M. E. Roberts S. M. M. Moser J. S. (2016). Deconstructing the emotion regulatory properties of mindfulness: an electrophysiological investigation. Front. Hum. Neurosci. 10:451. doi: 10.3389/fnhum.2016.00451, PMID: 27656139 PMC5013076

[ref51] Lindsay E. K. Creswell J. D. (2017). Mechanisms of mindfulness training: monitor and acceptance theory (MAT). Clin. Psychol. Rev. 51, 48–59. doi: 10.1016/j.cpr.2016.10.011, PMID: 27835764 PMC5195874

[ref52] Logsdon R. G. Teri L. (1997). The pleasant events schedule-AD: psychometric properties and relationship to depression and cognition in Alzheimer’s disease patients. The Gerontologist 37, 40–45. doi: 10.1093/geront/37.1.40, PMID: 9046704

[ref53] Losada A. Márquez-González M. Romero-Moreno R. López J. (2014). Development and validation of the experiential avoidance in caregiving questionnaire (EACQ). Aging Ment. Health 18, 897–904. doi: 10.1080/13607863.2014.896868, PMID: 24678984

[ref54] Manteau-Rao M. Barrows K. (2016). Caring for a Loved One With Dementia: A Mindfulness-Based Guide for Reducing Stress and Making the Best of Your Journey Together. 1st Edn. Oakland: New Harbinger Publications.

[ref55] Merrilees J. J. Bernstein A. Dulaney S. Heunis J. Walker R. Rah E. . (2020). The care ecosystem: Promoting self-efficacy among dementia family caregivers. Dementia (London, England) 19, 1955–1973. doi: 10.1177/1471301218814121, PMID: 30497302 PMC6541533

[ref56] Morris L. W. Morris R. G. Britton P. G. (1988). The relationship between marital intimacy, perceived strain and depression in spouse caregivers of dementia sufferers. Br. J. Med. Psychol. 61, 231–236. doi: 10.1111/j.2044-8341.1988.tb02784.x, PMID: 3179245

[ref57] Moskowitz J. T. Cheung E. O. Snowberg K. E. Verstaen A. Merrilees J. Salsman J. M. . (2019). Randomized controlled trial of a facilitated online positive emotion regulation intervention for dementia caregivers. Health Psychol. 38, 391–402. doi: 10.1037/hea0000680, PMID: 31045422 PMC6501812

[ref58] Neff K. (2003). Self-compassion: an alternative conceptualization of a healthy attitude toward oneself. Self Identity 2, 85–101. doi: 10.1080/15298860309032

[ref59] Ortner C. N. M. Corno D. Fung T. Y. Rapinda K. (2018). The roles of hedonic and eudaimonic motives in emotion regulation. Personal. Individ. Differ. 120, 209–212. doi: 10.1016/j.paid.2017.09.006

[ref61] Pinquart M. Sörensen S. (2003). Differences between caregivers and noncaregivers in psychological health and physical health: a meta-analysis. Psychol. Aging 18, 250–267. doi: 10.1037/0882-7974.18.2.250, PMID: 12825775

[ref001] Pinquart M. Sörensen S. (2004). Associations of caregiver stressors and uplifts with subjective well-being and depressive mood: a meta-analytic comparison. Aging Ment. Health 8, 438–449. doi: 10.1080/13607860410001725036 15511742

[ref62] Pinquart M. Sörensen S. (2006). Helping caregivers of persons with dementia: which interventions work and how large are their effects? Int. Psychogeriatr. 18, 577–595. doi: 10.1017/s1041610206003462, PMID: 16686964

[ref002] Pinquart M. Sörensen S. (2007). Correlates of physical health of informal caregivers: a meta-analysis. J. Gerontol. B. Psychol. Sci. Soc. Sci. 62, 126–137. doi: 10.1093/geronb/62.2.p126 17379673

[ref63] Pucciarelli G. Buck H. G. Barbaranelli C. Savini S. Simeone S. Juarez-Vela R. . (2016). Psychometric characteristics of the mutuality scale in stroke patients and caregivers. Gerontologist 56, e89–e98. doi: 10.1093/geront/gnw083, PMID: 27114475

[ref64] Quinn C. Clare L. Woods B. (2009). The impact of the quality of relationship on the experiences and wellbeing of caregivers of people with dementia: a systematic review. Aging Ment. Health 13, 143–154. doi: 10.1080/13607860802459799, PMID: 19347681

[ref65] Rabins P. V. Mace N. L. Lucas M. J. (1982). The impact of dementia on the family. JAMA 248, 333–335. doi: 10.1001/jama.1982.03330030039022 7087127

[ref66] Raes F. (2011). The effect of self-compassion on the development of depression symptoms in a non-clinical sample. Mindfulness 2, 33–36. doi: 10.1007/s12671-011-0040-y

[ref67] Riedijk S. R. De Vugt M. E. Duivenvoorden H. J. Niermeijer M. F. van Swieten J. C. Verhey F. R. J. . (2006). Caregiver burden, health-related quality of life and coping in dementia caregivers: a comparison of frontotemporal dementia and Alzheimer’s disease. Dement. Geriatr. Cogn. Disord. 22, 405–412. doi: 10.1159/000095750, PMID: 16966830

[ref68] Santorelli S. Florence Meleo-Meyer M. Koerbel L. Kabat-Zinn J. (2020). Mindfulness-Based Stress Reduction (MBSR) Authorized Curriculum Guide. [online] Available at: https://lotheijke.com/wp-content/uploads/2020/11/8-week-mbsr-authorized-curriculum-guide-2017.pdf

[ref69] Shaffer D. R. Dooley W. K. Williamson G. M. (2007). Endorsement of proactively aggressive caregiving strategies moderates the relation between caregiver mental health and potentially harmful caregiving behavior. Psychol. Aging 22, 494–504. doi: 10.1037/0882-7974.22.3.494, PMID: 17874950

[ref70] Siow J. Y. M. Chan A. Østbye T. Cheng G. H.-L. Malhotra R. (2017). Validity and reliability of the positive aspects of caregiving (PAC) scale and development of its shorter version (S-PAC) among family caregivers of older adults. The Gerontologist 57, gnw198–gnwe84. doi: 10.1093/geront/gnw198, PMID: 28082275

[ref71] Sörensen S. Conwell Y. (2011). Issues in dementia caregiving: effects on mental and physical health, intervention strategies, and research needs. Am. J. Geriatr. Psychiatry 19, 491–496. doi: 10.1097/jgp.0b013e31821c0e6e, PMID: 21502853 PMC3774150

[ref72] Spira A. P. Beaudreau S. A. Jimenez D. Kierod K. Cusing M. M. Gray H. L. . (2007). Experiential avoidance, acceptance, and depression in dementia family caregivers. Clin. Gerontol. 30, 55–64. doi: 10.1300/j018v30n04_04

[ref73] Spradley J. P. (1979). The Ethnographic Interview. Cambridge: Wadsworth Publishing Company.

[ref74] Team R. (2014). R: A Language and Environment for Statistical Computing. undefined. [online] Available at: https://www.semanticscholar.org/paper/R%3A-A-language-and-environment-for-statistical-Team/659408b243cec55de8d0a3bc51b81173007aa89b.

[ref75] THE WHOQOL GROUP (1998). Development of the World Health Organization WHOQOL-BREF quality of life assessment. Psychol. Med. 28, 551–558. doi: 10.1017/s0033291798006667 9626712

[ref76] Van Doesum N. J. Van Lange D. A. W. Van Lange P. A. M. (2013). Social mindfulness: skill and will to navigate the social world. J. Pers. Soc. Psychol. 105, 86–103. doi: 10.1037/a0032540, PMID: 23647176

[ref77] Wagner A. W. Logsdon R. G. Pearson J. L. Teri L. (1997). Caregiver expressed emotion and depression in Alzheimer’s disease. Aging Ment. Health 1, 132–139. doi: 10.1080/13607869757227

[ref78] Wong C. Merrilees J. Ketelle R. Barton C. Wallhagen M. Miller B. (2012). The experience of caregiving: differences between behavioral variant of frontotemporal dementia and Alzheimer disease. Am. J. Geriatr. Psychiatry 20, 724–728. doi: 10.1097/jgp.0b013e318233154d, PMID: 21941168 PMC4005886

[ref79] Yeager C. A. Hyer L. A. Hobbs B. Coyne A. C. (2010). Alzheimer’s disease and vascular dementia: the complex relationship between diagnosis and caregiver burden. Issues Mental Health Nurs. 31, 376–384. doi: 10.3109/01612840903434589, PMID: 20450339

