# Supplementary material for: A mindfulness-based intervention adapted to dementia caregivers: A study protocol for a randomized clinical control trial
Source: Front Psychol. 2022 Dec 20;13:1062452. doi: 10.3389/fpsyg.2022.1062452 (PMC9808397; doi:10.3389/fpsyg.2022.1062452)
Supplement: Supplementary file 1 [file Data_Sheet_1.docx]

Supplementary Material 1

# Interview Procedure

Caregivers will be asked the following questions via a semi-structured interview:

- Describe (name of patient) for me. What is he/she like?
- How do the two of you get along together?
- Describe something you enjoyed doing together
- Describe something challenging that you faced together
- What thoughts and feelings come up when you think about (name of patient)?
- Describe 1 or 2 of your daily activities?

# Lexicons

## Affective lexicon

According to the affective lexicon used by the program, there are approximately 1500 emotion words falling in two superordinate valence categories: positive and negative. Categories will be derived from 18 subordinate discrete categories of emotion as follows:

- 12 positive emotion categories: amusement, awe, content/relaxed, excitement/aroused, love/affection, pride, interest/preference, surprise.
- 15 negative emotion categories: aggressive, anger, contempt, disgust, embarrassment, fear, guilt, jealousy/envy, sadness/grief, shame

## Pronoun lexicon

- Me-Words: I, I’d, I’ll, I’m, I’ve, me, mine, my, myself
- You-Words: you, you’d, you’ll, you’re, you’ve, your, yours, yourself
- We-Words: our, ours, ourselves, us, we, we’d, we’ll, we’re, we’ve

## Rigidity lexicon

- Should-ing: should, must, have to/have got to, ought to, shouldn’t, hadn't, need to, suppose/supposed to, meant to, got to
- Never-Words: never, at no time, not at any time, not ever, not once, on no occasion, not at all, certainly not, not under/in any circumstances, under/in no circumstances, no way, no/not such, no matter, not in the least, not ever
- Always-Words: every time, each time, at all times, all the time, always, constantly, ever, consistently, forever, all

# Socioemotional Language Coding

Because the meaning of a word can be altered by its context, a team of coders will review each identified word to determine if it meets criteria for the selected categories. Additional contextual analysis will be performed to improve accuracy. Finally, the total number of words in each coding system will be divided by the total number of words spoken as well as the total number of coded words.

Each pronoun/rigidity word will be then placed into one of the following five categories. Only actual personal pronouns/ rigidity categories referring to the speaker, spouse, or couple will be used in narrative analyses.

- Actual (personal pronouns/ rigidity words referencing the speaker, other spouse, or couple)
- Disfluencies (pronouns/ rigidity words used prior to a repetition and/or the truncation of a proposition).
- Generic (pronouns/ rigidity words referring to a general or universal other).
- Filler (pronouns/ rigidity words used as part of an idiomatic phrase used to fill a speech pause).
- No code (pronouns/ rigidity words used in references to the speech of a third person).
